# Synthesis of Graphene Oxide Based Sponges and Their Study as Sorbents for Sample Preparation of Cow Milk Prior to HPLC Determination of Sulfonamides

**DOI:** 10.3390/molecules24112086

**Published:** 2019-05-31

**Authors:** Martha Maggira, Eleni A. Deliyanni, Victoria F. Samanidou

**Affiliations:** 1 Laboratory of Analytical Chemistry, Department of Chemistry, Aristotle University of Thessaloniki, GR-541 24 Thessaloniki, Greece; marthamaggira@gmail.com; 2 Laboratory of General and Environmental Technology, Department of Chemistry, Aristotle University of Thessaloniki, GR-541 24 Thessaloniki, Greece; lenadj@chem.auth.gr

**Keywords:** sulfonamides, HPLC, graphene oxide, sponge, milk

## Abstract

In the present study, a novel, simple, and fast sample preparation technique is described for the determination of four sulfonamides (SAs), namely Sulfathiazole (STZ), sulfamethizole (SMT), sulfadiazine (SDZ), and sulfanilamide (SN) in cow milk prior to HPLC. This method takes advantage of a novel material that combines the extractive properties of graphene oxide (GO) and the known properties of common polyurethane sponge (PU) and that makes sample preparation easy, fast, cheap and efficient. The PU-GO sponge was prepared by an easy and fast procedure and was characterized with FTIR spectroscopy. After the preparation of the sorbent material, a specific extraction protocol was optimized and combined with HPLC-UV determination could be applied for the sensitive analysis of trace SAs in milk. The proposed method showed good linearity while the coefficients of determination (R^2^) were found to be high (0.991–0.998). Accuracy observed was within the range 90.2–112.1% and precision was less than 12.5%. Limit of quantification for all analytes in milk was 50 μg kg^−1^. Furthermore, the PU-GO sponge as sorbent material offered a very clean extract, since no matrix effect was observed.

## 1. Introduction

Sulfonamides are a group of synthetic antibacterial agents, which are widely used in veterinary practice for prophylactic and therapeutic purposes and as feed additives. Due to their ability to inhibit folic acid synthesis in microorganisms, they are commonly used against a wide range of bacteria, protozoa, parasites, and fungi [1,2,3].

However, the improper administration of sulfa drugs in dairy husbandry and the insufficient withdrawal periods can lead to noncompliant residues in animal originated foods, a fact which can contribute to several concerns in the dairy industry and public health [4].

In humans, such concerns comprise the rise of allergic or toxic reactions and the development of drug-resistance, whereas in the dairy industry they provoke the inhibition of bacterial fermentation in cheese and yoghurt production [5]. In order to safeguard public health and ensure food safety, monitoring of such residues in products designated for human consumption is considered mandatory. For this reason, the European Union has established a maximum residue level (MRL) for sulfonamides in foodstuffs of animal origin, which in the case of milk is 100 μg kg^−1^ [6]

Additionally, several methods have been described for the detection and/or determination of sulfonamides in foods of animal origin such as microbial inhibition assays, immunochemical methods, capillary electrophoresis (CE), gas chromatography (GC), and HPLC [5,7].

Sample preparation is a key step prior to the detection of sulfonamides present in different kinds of samples. The clean-up procedure of various matrices can be accomplished by either traditional techniques, such as liquid-liquid extraction (LLE) [8], or modern methods, like solid phase extraction (SPE) [9], solid phase micro extraction (SPME) [1,10], fabric phase solid extraction [11], matrix solid phase dispersion (MSPD) [12] and Quick, Easy, Cheap, Effective, Rugged and Safe (QuEChERS) method [13,14]. Most of the aforementioned techniques depend on an absorbent material to achieve high analytical specificity and selectivity.

However, in the analysis of complex matrices, many innovative materials have emerged as valuable tools to enhance the efficiency of the extraction and isolation of the target analytes. As such, graphene-based materials are preferred to other carbon-based nanomaterials due to their great potential on the sample preparation procedure. Graphene (G) is a two dimensional nanomaterial with extraordinary physicochemical properties such as thermal and chemical stability, thermal conductivity, hydrophobicity, and large specific surface area [15]. Graphene oxide (GO) is a single-atomic layered material, an important derivative of graphene with similar structure, which is composed easily from the oxidation of graphite. However, GO is more polar than G because of the hydroxyl (–OH) and carboxyl (–COOH) groups, a characteristic that facilitates GO bonds into other compounds such as aminopropyl silica [16].

Graphene based materials are extensively applied in SPE procedure as they offer high sorption efficiency for organic compounds and metal ions mainly in environmental samples [17,18,19]. Although G and GO demonstrate excellent sorbent characteristics, many limitations have been reported concerning their isolation from well dispersed solutions and their sheets’ restacking or escaping from the SPE column [20,21].

In order to surpass the problems having occurred during the elution and sample loading in SPE, new sample preparation techniques have been developed such as the use of graphene-based materials in dispersive solid phase extraction (DSPE) and MSPD. In DSPE the absorbent is mainly utilized in food [22] and environmental samples [23,24,25,26], whereas MSPD has been performed for the extraction of sulfonamides in milk samples [27].

Recently, melamine sponge was functionalized with graphene, via a microwave-assisted hydrothermal process, in order to be used as adsorbent for SAs extraction from milk, egg, and environmental water [28]. The proposed method was highly accurate and sensitive for the analysis of nine SA’s. However, it is not referred to the determination of sulfathiazole (STZ), sulfamethizole (SMT), and sulfanilamide (SN). In the current study, commercial polyurethane (PU) sponges, a kind of cheap porous material, were examined for SAs extraction from milk. PU sponges, compared with other sponge materials, such as melamine [29,30], and chitosan sponge [31] present certain advantages like easy access, low cost, and high resilience, excellent flexibility, and reuse [32]. Moreover, the surface of the PU sponge was used as a skeleton for hydrophobic modifiers. Hence, in the current study, surface modification was achieved via a green route at ambient conditions.

Polyurethane (PU) sponges with a unique 3D structure have a potential application as absorbents due to their advantages of easy access, low cost, and high resilience compared to other porous materials, such as melamine foam and chitosan sponge. Although PU sponge is hydrophilic, modifications or physical coating like functionalization with graphene are required to increase the hydrophobicity and are usually used to achieve higher efficiency in separations [32].

Consequently, the objective of this study was to combine the unique properties of PU sponge being functionalized with GO in order to serve as an innovative absorbent material in the sample preparation procedures. Due to its properties of low cost, time saving, and simplicity, the GO-PU material was further used for the determination of sulfonamides in cow milk samples prior to HPLC-DAD method.

## 2. Results and Discussion

### 2.1. Characterization

Polyurethane sponge was used as a base material in order to be functionalized with graphene oxide. Polyurethane presents an open-hole structure, with a high porosity as well as a rich surface chemistry with surface-groups that can attract and react with different molecules. Graphene oxide was embodied in the PU skeleton after the dispersion of GO in water. Graphene oxide was connected to polyurethane after chemical interactions between the GO (epoxy-groups) and polyurethane surface groups (C=O and –N–H groups). After the polyurethane functionalization with graphene oxide, the sponge prepared appeared with a black color and presented hydrophobicity that was further increased after the coating with PVA.

**Figure 1:**
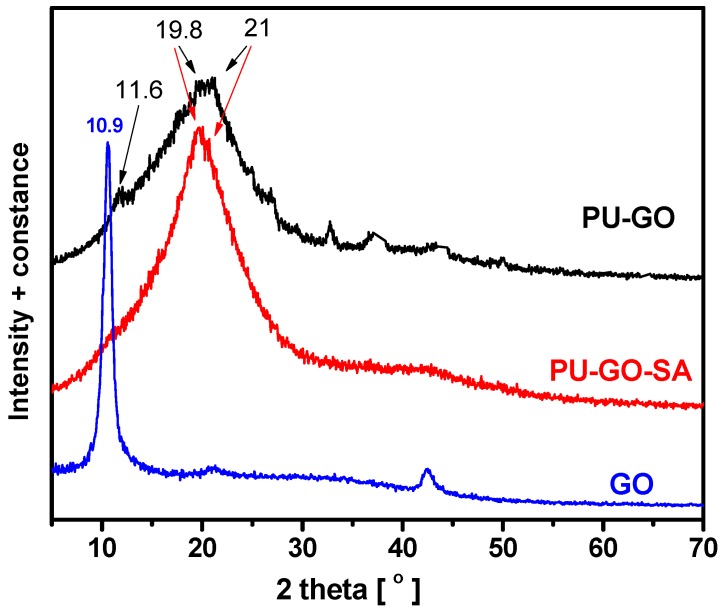
X-ray diffraction (XRD) patterns of the graphite oxide (GO), the graphene oxide impregnated sponge (PU-GO), and the sponge after the adsorption of sulfonamides (PU-GO-SA).

**Figure 2:**
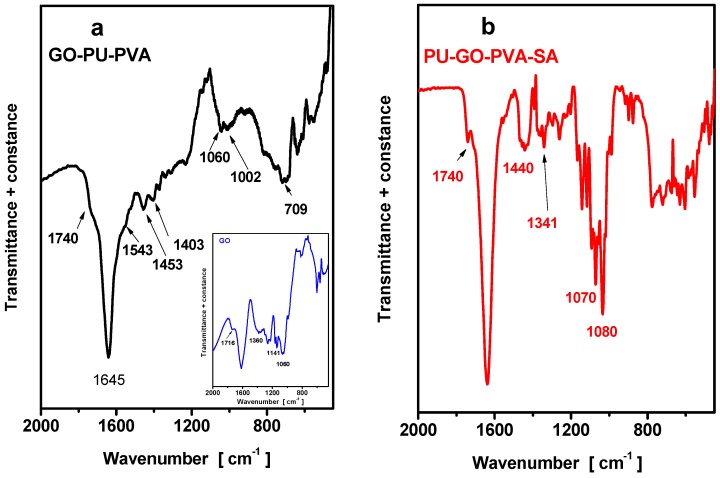
Fourier-transform infrared (FTIR) spectra for (**a**) polyurethane-graphene oxide- polyvinyl alcohol (PU-GO-PVA) sponge raw and after (**b**) the absorption of sulfonamide’s (SA’s) (PU-GO-PVA-SA)-(in the inset the spectrum of GO).

The XRD diffraction patterns of the prepared graphite oxide (GO) as well as of the GO impregnated sponge before (PU-GO) and after the PVA coating (PU-GO-PVA) are presented in Figure 1. Graphite presents a sharp diffraction peak at 26.6° in the XRD pattern (not presented), attributed to interlayer (002) spacing (d = 0.33 nm). Τhe characteristic XRD peak of graphite oxide appeared at 2θ = 10.9°; as estimated by the Bragg’s law, the interlayer distance between the carbon layers, increased from 0.33 nm for graphite to 0.81 nm for GO [33]. In the XRD pattern of the GO impregnated sponge (PU-GO) the characteristic XRD peak of graphite oxide, at 2θ = 10.9°, was not present, indicating that the layered structure of GO was destroyed. A diffraction peak at 2θ = 21° could be due to PVA while the broad peaks at around 11.6° and 19.8° indicated some degree of crystallinity of the PU [34,35,36]. The XRD pattern for the sample after the sulfonamide adsorption (PU-GO-SA), which is also presented in Figure 2, reveals that a decrease of crystallinity was observed, evidenced by the disappearance of the peak at 2θ = 11.6°.

FTIR spectroscopy was used in this study to identify the possible interactions between GO and PU (PU-GO), between PU-GO and PVA (PU-GO-PVA sponge) as well as between the sponge and the sulfonamides (PU-GO-PVA-SA) in order for the adsorption mechanism to be revealed. The FTIR spectra of PU-GO-PVA as well as of PU-GO-PVA after the sorption of sulfonamide (PU-GO-PVA-SA), are presented in Figure 2. The FTIR spectra of GO is presented in the inset of Figure 2. GO contains polar groups on the edges of graphite layers such as carbonyl, carboxyl, and epoxide, as well as hydroxyl groups within the basal planes of the graphene sheets. In the spectrum of GO (Figure 2a), the bands at 1050–1100 cm^−1^ and ~1716 cm^−1^ can be attributed to carboxylic groups whereas the band at ~1600 cm^−1^ can be attributed to C=C stretching mode of the sp^2^ carbon skeletal network and/or to epoxy groups. The band at 1356 cm^−1^ is due to C–OH stretching of O–H groups, while the band at 1045 and at 1141 cm^−1^ can be also attributed to epoxy and alkoxy C–O groups, respectively.

Polyurethane (PU) is a polymer obtained after the polymerization of diisocyanate and polyol that contains C=O and –NH groups (electron donating sites); these groups are able to form hydrogen bonds with graphene oxide during the complexation. The spectra of PU-GO-PVA sponge presented peaks at 1740 and 1060 cm^−1^ attributed to carboxyl and epoxy groups, respectively, at a lower intensity compared to the relative peaks of the spectra of GO, indicating the involvement of these groups in the composite synthesis. The peaks at 1543 cm^−1^ could be attributed to amide II formation after reaction of the carboxylic groups of GO with –NH groups of PU while the peaks at about 1453 cm^−1^ could be attributed to –CH_3_ groups of PVA indicating the covering [37,38,39].

The most significant spectra alterations for the GO-PU-PVA after the SA adsorption (GO-PU-PVA-SA sample), are the new bands appearing at 1260 and 1070 cm^−1^ in addition to the diminishing of the peaks at 1191, 1130 and 1740 cm^−1^ (carbonyl) absorption bands (Figure 2). The new band at 1440 cm^−1^ can be attributed to amide I formation due to interactions between the SA amines and the sponge carboxylates, causing the diminishing of the band at 1740 cm^−1^. The new band at 1260 cm^−1^, can be attributed to hydrogen bond interaction between the GO-PU-PVA carboxyl groups and the sulfones/O=S=O groups of SA which are strong hydrogen-bond acceptors. It is obvious that the grafting of PU with extra carboxyl groups enhanced the SA adsorption owning to their reactions with the amines and the hydrogen bond with the sulfones/O=S=O groups of the SA. This was also reported for dorzolamine encapsulation to chitosan, as well as for pramipexole adsorption on activated carbon.

### 2.2. Synthesis Optimization

**Figure 3:**
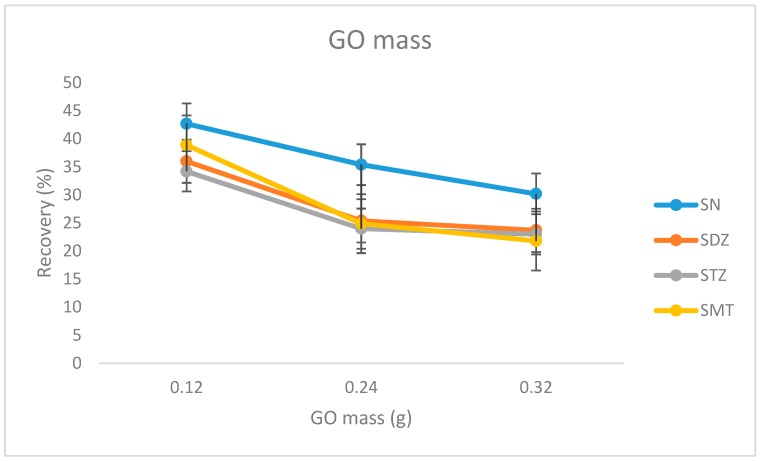
Effect of the graphene oxide (GO) mass on the adsorption efficiency of the sulfonamides.

The mass of the material retained in the sponge was initially studied, keeping its second mass at 0.04 g. After selecting three different levels (0.12, 0.24, and 0.32 g), the procedure of the sponge preparation was followed. The sample preparation was performed in standard solutions with all three materials. From the results as presented in Figure 3, it seems that the mass of 0.12 g is more effective for the adsorption.

The size of the sponge was optimized after the testing of two different sizes. Particularly 0.04 g and 0.07 g sponge were dipped in the dispersed solution. The results showed that the bigger sponge is sufficient to achieve the optimum adsorption.

**Figure 4:**
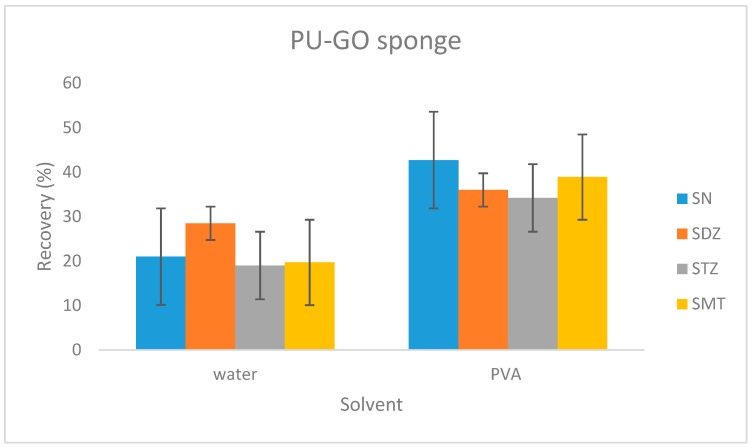
Effect of the solvent in the absolute recoveries of the four sulfonamides.

For the PU-GO sponge formation, the GO molecules should be immobilized during its preparation. This is accomplished with the adding of a solvent like water or some polymer, of which polyvinyl alcohol (PVA) is more common due to its low cost. In the present research, two such solvents were tested, water and PVA. As shown in the results (Figure 4), PVA helps in the sample preparation procedure.

Different solutions of NH_3_/EtOH containing 60 mL of the mixture were prepared in three different volume ratios (4:1, 1:1 and 1:4) and were further applied in the functionalization of the GO-PU material. The results revealed that the quantity of NH_3_ was crucial to the absorption and that the volume ratio 4:1 achieved higher efficiency.

### 2.3. Chromatography

**Figure 5:**
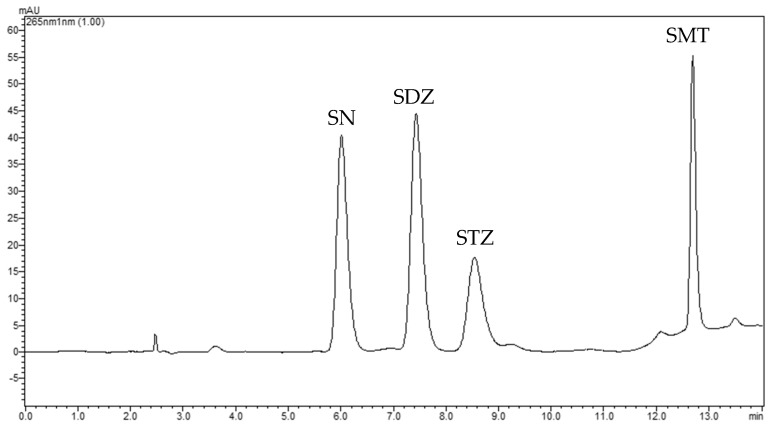
A typical HPLC chromatogram of standard solution of examined analytes at the concentration of 5 ng μL^−1^. Peaks are as follows: SN: 6.345 min, SDZ: 7.566 min, STZ: 8.748 min, and SMT: 12.899 min.

The target analytes were separated by gradient elution. Optimum gradient program was chosen as providing good analytes’ resolution, at the shortest analysis. A typical chromatogram is shown in Figure 5. The retention times were observed at 6.345, 7.566, 8.748, and 12.899 min for SN, SDZ, STZ, and SMT respectively.

### 2.4. Sample Preparation Optimization

All initial optimization experiments were performed using standard solutions of sulfonamides. The optimum conditions established were further checked for their appropriateness to the milk matrix.

In the loading and elution step different methods were tested. Although stirring showed the best results in the tests with the standard solutions, as shown in Table 1 the extraction declined sharply when the milk samples were tested and the recovery rates ranged from 7 to 14%. Thus, centrifugation in low rates was selected. Centrifugation at low rates had two purposes: (1) sufficient sample interaction with the material, and (2) preventing the adsorbent from escaping from the structure of the sponge. High centrifugation rates hindered the extraction process. With regards to sonication, GO particles were released from the sponge and sample handling was difficult.

Additionally, the volume of the sample, the elution solvents, the size of the sponge, loading and elution time, and the pH were optimized. The extraction was conducted with two different volume samples (1.5 and 3 g) that were spiked with the same amount of the target analytes. The results revealed a decrease in the extraction efficiency by increasing the volume of the sample.

With regards to the elution, methanol (MeOH) and acetonitrile (ACN) were tested both separately and in mixture. It is obvious from the results that the mixed solution increases the efficiency of the elution. In order to succeed better results, 1% acetic acid was added. The addition of acetic acid was successful and the optimum volume ratio for the CH_3_COOH/ACN/MeOH solution was 50:40:10.

As for the loading and elution time 10, 15, 20 min were tested. From the results it is observed that 10 min are not enough for the loading and the extraction of the target analytes. However, 15 and 20 min yielded similar results, and the shortest time was selected to reduce the process time.

The effect of the pH in the extraction efficiency was tested, adding 0.5 mL of buffer solution into the sample. Table 2 presents the results obtained from the addition of pH 3, 5, 7, and 9 buffer solution in milk sample. It is obvious from the results that the optimum pH is 5, whereas lower or higher pH values results in decrease in the adsorption for all SAs.

**Figure 6:**
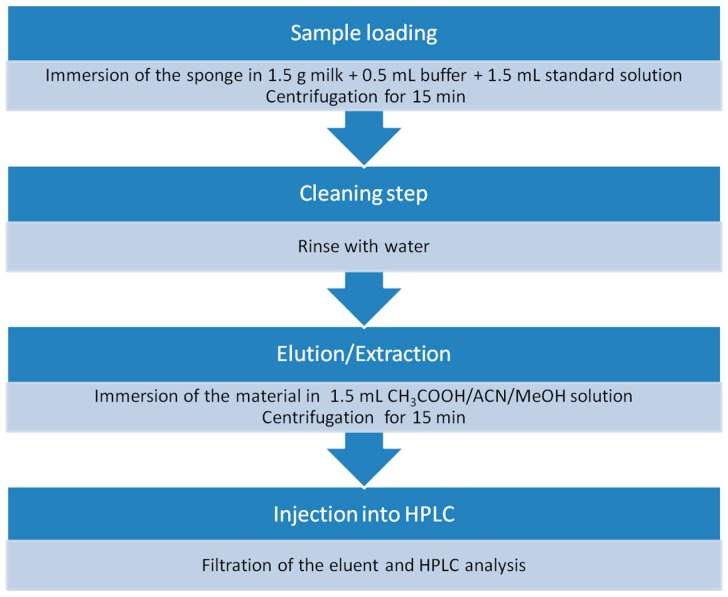
Steps of sample preparation procedure.

**Figure 7:**
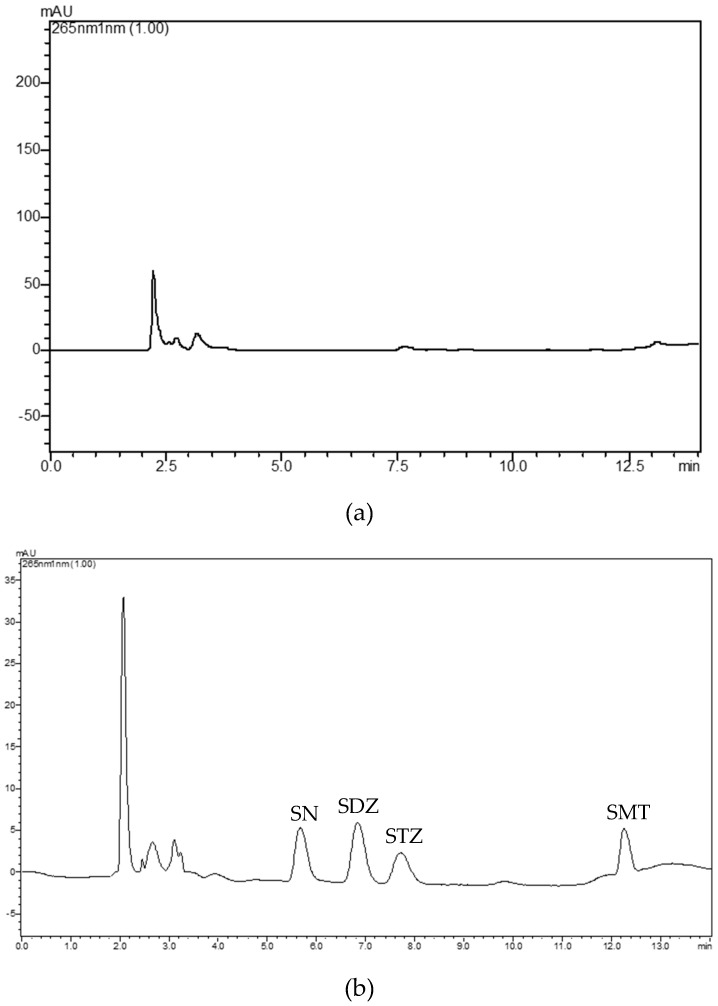
Chromatogram of (**a**) blank milk sample and (**b**) spiked milk sample at a concentration of 300 μg kg^−1^.

The proposed sample preparation protocol is very simple and rapid, with low consumption of organic solvents and very clean background signal. Figure 6 illustrates the simple pretreatment procedure. Typical chromatograms of a blank and a spiked milk sample are shown in Figure 7a,b. It is clear that the peaks of the substrate do not interfere with the analysis as they elute at different times.

### 2.5. Method Validation

#### 2.5.1. Selectivity

The good resolution between the chromatographic peaks of analytes and the absence of interferences in the spiked milk samples indicate that a good selectivity was achieved.

#### 2.5.2. Linearity and Sensitivity

Standard solutions showed linearity for all of the target analytes within the range of 0.5 to 10 ng μL^−1^ and showed and good correlation coefficients (0.981–0.999). Moreover, calibration curves were constructed using fortified milk samples after sample preparation, and good coefficients of determination between 0.9969 and 0.999 were achieved over the examined range. (Table 3). Limit of quantification for all analytes in milk was 50 μg kg^−1^.

#### 2.5.3. Precision and Accuracy

The precision of the method was based on within-day repeatability and between-day precision. The former was assessed by replicate (*n* = 4) measurements from a spiked milk sample at the MRL level for all examined sulfonamides. The recoveries of spiked samples were calculated by comparison of the peak area ratios for extracted compounds toward the values derived from spiked calibration curves. In Between-day reproducibility a triplicate determination was performed for a period of three days (Table 4). Precision and accuracy was determined at three concentration levels according to the 657/2002/EC decision [40].

#### 2.5.4. Decision Limit and Capability of Detection

Decision limit (CCα) is defined as “the limit at and above which it can be concluded with an error probability” and it was calculated after the analysis of 20 spiked milk samples at the MRLs of each compound. The decision limits CCa were 100.2 μg kg^−1^ for SN, 100.3 μg kg^−1^ for SDZ, 100.4 μg kg^−1^ for STZ, and μg kg^−1^ for 100.3 SMT. Capability of detection (CCb) defined as ‘‘the smallest content of the substance that may be detected, identified, and/or quantified in a sample with an error probability of b’’ and it was calculated after the spiking of 20 blank milk samples at the CCa level of each compound. The capability of detection (CCb) were 110.7 μg kg^−1^ for SN, 109.3 μg kg^−1^ for SDZ, 115.4 μg kg^−1^ for STZ, and 114.3 μg kg^−1^ for SMT.

### 2.6. Application to Real Samples

The method was applied for the determination of the examined analytes in cow milk samples from local food stores. Five random samples of three different types of milk were collected and analyzed, including full-fat (3.5%), semi-skimmed (1.5%), and skimmed (0%) milk. All analyzed samples were negative in the presence of examined analytes.

### 2.7. Comparison with Other Methods

The method described in this study was compared with previous analytical approaches for the determination of SAs in milk. The analysis’ results are comparable with those attained by other methods, with fairly good recoveries and quite satisfactory sensitivity. Although it provides higher LODs and LOQs than previously reported methods, it is a less costly (no commercial SPE products are needed) and less time-consuming method with easy handling of sponge and does not require highly sophisticated equipment since no MS is used (Table 5).

## 3. Materials and Methods

### 3.1. Chemicals and Reagents

Sulfathiazole (STZ), sulfamethizole (SMT), sulfadiazine (SDZ), and sulfanilamide (SN) were purchased from Sigma-Aldrich (Steinheim, Germany). HPLC grade acetonitrile and methanol obtained from Chem-Lab (Zedelgem, Belgium). Formic and acetic acid were of analytical grade and purchased from Chem-Lab (Zedelgem, Belgium) and Merck (Darmstadt, Germany) respectively. Ethanol, reagent grade (Chem-Lab, Zedelgem, Belgium) and ammonia, 25% solution (PANREAC QUIMICA SA, Barcelona, Spain) were used for the sponge optimization. Polyvinyl alcohol high molecular weight solid, (PVA 98–99 hydrolized) was purchased from A Johnson Company (New Brunswick, NJ, USA).

Graphite was purchased from Sigma Aldrich (St. Louis, MO, USA). Double-deionized water was filtered with 0.45 μm filter membrane before use.

Milk samples were collected from local market (Thessaloniki, Greece). Different fresh milk types were analyzed including skimmed (0% fat), semi-skimmed (1.5% fat), and full-fat milk (3.5% fat). All milk samples were kept refrigerated (at 4 °C) until use.

### 3.2. Instrumentation

Chromatographic separation and analysis were carried out on a Shimadzu HPLC system coupled to a Diode Array Detector (DAD) (Kyoto, Japan), equipped with Rheodyne 7725i 20 μL loop (Cotati, CA, USA). The system consisted of a Shimadzu LC-10 ADVP pump and a Shimadzu FCV-10ALVP solvent mixer (Kyoto, Japan). The chromatographic separation was achieved using a Merck-Lichrospher RP8e, 5 μm 250 × 4 mm analytical column (Darmstadt, Germany). Degassing of the mobile phase was performed by helium DGU-10B degassing unit by Shimadzu (Kyoto, Japan) directly in the solvent reservoirs. The system was controlled by Shimadzu LabSolutions software (Shimadzu, Kyoto, Japan) which was also used for the data acquisition and analysis.

A glass vacuum filtration apparatus obtained from Alltech Associates (Deerfield, IL, USA), was employed for the filtration of the solvents using cellulose nitrate 0.2 μm membrane filters from Whatman (Maidstone, UK) prior to use. A Glasscol Vortexer (Terre Haute, IN, USA), an ultrasonic bath Transonic 460/H (Elma, Germany), a Reacti-Vap evaporator model from PIERCE (Rockford, IL, USA), and a Hermle centrifugation (Gosheim, Germany) were acquired for the sample preparation. Moreover, a 20–200 μL micropipette ISOLAB Laborgerate GmbH (Wertheim, Germany) was used for the preparation of the standard solutions.

XRD measurements were performed on a Philips PW1820 X-ray diffractometer. The Fourier Transform Infrared Spectra (FTIR) were measured on a Nicolet 560 (Thermo Fisher Scientific Inc., MA, USA) spectrometer.

### 3.3. Chromatography

The mobile phase consisted of water, containing 0.1% (*v*/*v*) formic acid (A), acetonitrile (B), and methanol (C). The analytes were separated following a gradient elution program, starting at 80:3:17 (*v*/*v*/*v*), turning to 74:6:20 (*v*/*v*/*v*) in the next 7.5 min, kept isocratic for 2.5 min, and finally changing to 50:10:40 (*v*/*v*/*v*) in the last three minutes. The flow rate was set at 1.0 mL min^−1^, while monitoring of the analytes was set at 265 nm.

### 3.4. Functionalization of Sponges

**Figure 8:**
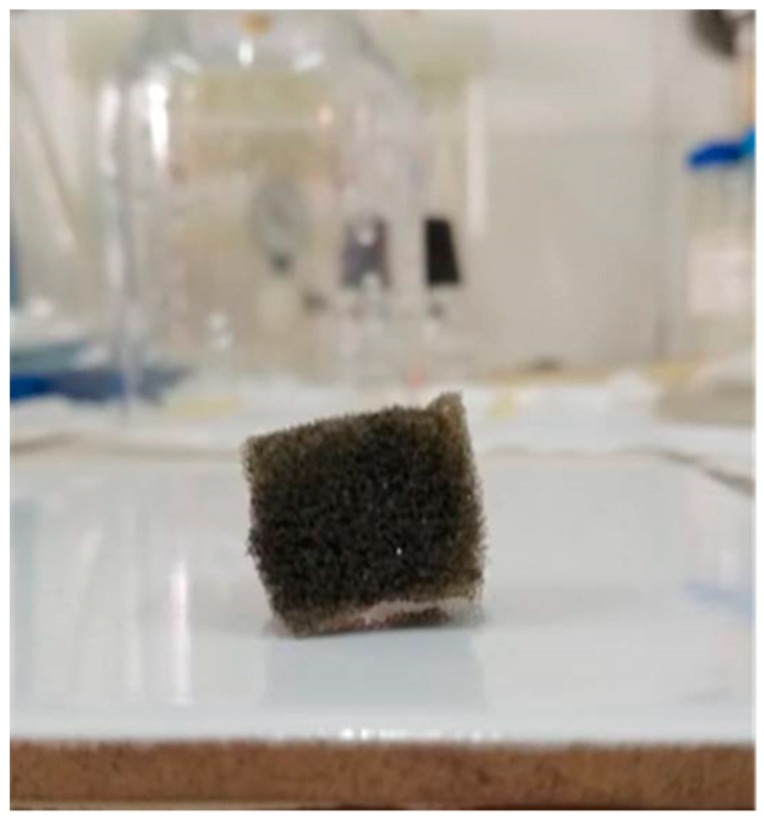
Image of the polyurethane-graphene oxide (PU-GO) sponge.

A commercially available polyurethane sponge was cut into cubes, immersed into ethanol/water solution, and placed in an ultrasonic bath for 20 min. The sponge was left at room temperature to dry and then it was dipped in a GO mixture for 24 h to be stirred mechanically. The mixture was prepared by the addition of 0.12 g GO in 60 mL NH_3_/EtOH solution (4:1, *v*/*v*). When mechanical stirring was completed, the sponge was left to dry in room temperature. Subsequently it was rinsed with water and PVA solvent was added as a final step. The PU-GO sponge is shown in Figure 8.

### 3.5. Sample Preparation

In the present study, defatted bovine milk was used and the proteins’ precipitation was achieved by adding 3 mL of ACN in 1.5 g of milk. The pH was adjusted to 5.0 by using 0.5 mL of buffer solution (70% CH_3_COONa 0.2 M/30% CH_3_COOH 0.2 M). The sponge was initially placed in a vial containing 1.5 g of milk and the system was centrifuged at low rpm for 15 min. The material was rinsed with deionized water and then squeezed to wash the water away. Subsequently, 1.5 mL of 1% CH_3_COOH/ACN/MeOH solution (50:40:10 *v*/*v*/*v*) was added to the sponge and the analytes were eluted by centrifugation at low rpm for 15 min. The eluent was filtered and injected in the HPLC column.

In the case of fat containing milk samples, centrifugation was applied for fat removal prior to deproteinization. Moreover, sample preconcentration was applied by evaporation of elution solvent prior to HPLC analysis and reconstitution to 100 μL when necessary and in order to reach the legislation demands.

### 3.6. Standard Solution Preparation

For the chromatographic analysis, stock standard solutions of each analyte were prepared at a concentration of 100 ng μL^−1^ using a solvent with the same composition as the mobile phase. Stock standard solutions were stable for six months at 4 °C, while working standards were prepared on a daily basis. The calibration curves were constructed by the use of solutions being prepared at concentrations of 0.5–10 ng μL^−1^.

### 3.7. Method Validation

The method was validated using spiked samples, under the optimal conditions, in terms of linearity, sensitivity, selectivity, and precision (repeatability and between-day precision), decision limit (CCa), decision capability (CCb), and stability according to the European Decision 657/2002/EC [40].

Linearity was studied by triplicate analysis of working standard solutions at concentration levels between 0.5 ng μL^−1^ to 10 ng μL^−1^. In milk, linearity was examined by triplicate analysis of spiked samples within the range of 50 μg kg^−1^–10,000 μg kg^−1^ and calibration curves were calculated. Limits of detection (LOD) and quantification (LOQ) were considered as the concentration giving a signal to noise ratio of 3 and 10, respectively. The selectivity of the method was proved by the absence of interference of endogenous compounds in the analysis of blank milk samples.

Precision and accuracy were calculated by analyzing spiked samples at the concentration levels of 50 μg kg^−1^, 100 μg kg^−1^ and 150 μg kg^−1^, which correspond to the ½ MRL, MRL, and 1 ½ MRL of sulfonamides [6]. Within-day repeatability was examined by 4 measurements at the above concentration levels. Between-day precision was assessed by performing triplicate analysis at the same concentration levels in three days. The relative recovery was calculated using the formula of the percentage of the ratio of the analyte mass that was found in the spiked sample, to the spiked mass.

Decision limit (CCa) was calculated using the equation CCa = MRL + 1.64 × SD, where SD is the standard deviation of the duplicate measurements of twenty milk samples spiked at MRL concentrations of each analyte. Decision capability (CCb) was calculated using the equation CCb = CCa + 1.64 × SD, with the SD being the standard deviation of the duplicate measurements of twenty milk samples spiked at CCa concentrations of each sulfonamide.

## 4. Conclusions

In the present study a new novel material was presented. Particularly, a PU-GO sponge was prepared, taking advantage of the unique properties of GO combined with the characteristics of the common PU sponge. This novel material was applied for the sample preparation of milk samples for the determination of sulfonamides prior to HPLC. The easy preparation of the material and the extremely fast, simple, and green sample preparation procedure make the proposed method suitable for the analysis of a complex matrix such as milk. It is the first time that the PU-GO sponge was applied for the determination of sulfonamides in milk samples. Furthermore, it is a less costly and time-consuming method and requires less equipment than previously reported methods.

## Figures and Tables

**Table 1:** Effect of the loading/elution time and the extraction procedure on the efficiency of the method. (SN = sulfanilamide, SDZ = sulfadiazine, STZ = sulfathiazole, SMT = sulfamethizole)

Loading/Elution Time (min)		Absolute Recovery Rates (R%)			
		SN	SDZ	STZ	SMT
Rest	15/15	21.1	23.5	29.9	29.3
Sonication	7/7	15.6	21.2	29.7	33.7
Stirring	15/15	22.3	28.7	34.8	36.4
Centrifugation	15/15	30.9	24.0	27.6	29.6

**Table 2:** Effect of the pH on the adsorption efficiency of the four sulfonamides. (SN = sulfanilamide, SDZ = sulfadiazine, STZ = sulfathiazole, SMT = sulfamethizole). Optimum pH value is given in bold.

	Absolute Recovery Rates (R%)			
pH	SN	SDZ	STZ	SMT
3	21.8	22.0	31.7	29.3
**5**	**22.2**	**27.5**	**36.1**	**31.7**
7	12.3	17.1	21.7	17.2
9	15.0	22.0	27.9	19.0

**Table 3:** Linearity data in standard solutions and spiked milk samples. (SN = sulfanilamide, SDZ = sulfadiazine, STZ = sulfathiazole, SMT = sulfamethizole).

Analytes	Calibration Curve	Coefficients of Determination (R^2^)
	Standard Solutions	
SN	y = 119367x + 366.66	0.999
SDZ	y = 121371x + 27142	0.995
STZ	y = 64281x + 16245	0.999
SMT	y = 58218x + 13560	0.981
	**Milk**	
SN	y = 14785x − 1957.4	0.996
SDZ	y = 17012x − 3034.3	0.998
STZ	y = 10182x − 2023.9	0.991
SMT	y = 8489.3x − 2609.2	0.991

**Table 4:** Precision and accuracy parameters of the method for the determination of sulfonamides in milk samples. (SN = sulfanilamide, SDZ = sulfadiazine, STZ = sulfathiazole, SMT = sulfamethizole).

Added Concentration (μg kg^−1^)	Analyte	Intra-Day *n* = 4		Inter-Day *n* = 3 × 3	
		R%	RSD	R%	RSD
50	SN	98.2	7.6	97.6	7.1
	SDZ	106.7	6.9	104.3	3.3
	STZ	93.6	8.5	95.6	9.8
	SMT	93.4	10.4	90.8	11.0
100	SN	103.3	4.0	107.7	4.0
	SDZ	112.1	10.8	105.3	0.4
	STZ	96.8	11.0	90.2	10.9
	SMT	92.8	11.8	97.6	12.4
150	SN	100.2	10.4	102.4	7.6
	SDZ	108.7	3.0	100.1	6.0
	STZ	96.6	9.8	92.8	12.0
	SMT	101.7	10.3	95.3	9.5

**Table 5:** Performance of the presented method in comparison with previously reported analytical methods.

Analytes	Sample Preparation	Analytical Technique	Run Time (min)	LOD-LOQ	Recovery (%)	Ref
4SAs	MSPE	HPLC-AS	N/A	LOD (ng/mL): 2.0–2.5 LOQ (ng/mL): 6.0–7.5	92–105	[41]
38 veterinary drugs (18SAs)	SPE	UHPLC-ESI-MS/MS	13.5	CCα (µg/kg): 109–114 (SAs) CCβ (µg/kg): 116–123 (SAs)	87–119 (all analytes)	[42]
6SAs	SPE	HPLC-DAD	15.3	LOD (µg/kg): 1.9–13.3 LOQ (µg/kg): 5.6–42.2	N/A	[7]
9 SAs	MSPE	HPLC-DAD	35	LOD (µg/L): 7–14	81.8–114.9	[27]
5 SAs	MSPE	HPLC-UV	8	LOD (µg/L): 1.16–1.59 LOQ (µg/L): 3.52–4.81	62.0–104.3	[12]
SMZ, SIX and SDMX	FPSE	HPLC-UV	6.5	CCα (µg/kg): 114.4–116.5 CCβ (µg/kg): 104.1–118.5	93–107	[11]
9 SAs	GMeS microextraction	HPLC-DAD	30	LOQ (µg/kg): 0.31–0.91	90–105	[28]
4 SAs	PU-GO sponge microextraction	HPLC-DAD	14	LOQ: 50 (μg/kg)	90.2–112.1	This study
